# Ensemble deep learning architectures for detecting pulmonary tuberculosis in chest X-rays

**DOI:** 10.1038/s41598-025-30792-x

**Published:** 2026-01-09

**Authors:** Alba García Seco de Herrera, Ekin Yagis, Nichapat Pinpo, Vahid Abolghasemi, Jarutas Andritsch, Sitthichok Chaichulee, Yashin Dicente Cid, Thammasin Ingviya

**Affiliations:** 1 UNED, Madrid, Spain; 2 Department of Surgery & Cancer, Faculty of Medicine, Imperial College London, London, UK; 3 Department of Clinical Research and Medical Data Science, Faculty of Medicine, Prince of Songkla University, Songkhla, Thailand; 4 School of Computer Science and Electronic Engineering, University of Essex, Colchester, UK; 5 School of Technology and Maritime Industries, Southampton Solent University, Southampton, UK; 6 Department of Biomedical Sciences and Biomedical Engineering, Faculty of Medicine, Prince of Songkla University, Songkhla, Thailand; 7 Roche Diagnostics, Sant Cugat del Vallès, Spain; 8 Department of Family and Preventive Medicine, Faculty of Medicine, Prince of Songkla University, Songkhla, Thailand

**Keywords:** Convolutional neural networks (CNNs), Ensemble deep learning, Pulmonary tuberculosis detection, Chest radiography, Medical image analysis, Respiratory disease screening, Diagnostic markers, Computer science, Biomedical engineering

## Abstract

Tuberculosis (TB) remains a major global health challenge, causing approximately 1.4 million deaths annually. In many high-burden regions, limited access to expert radiological interpretation leads to delayed or missed diagnoses. To address this, we propose a cost-effective, automated TB screening method suitable for under-resourced settings. Our method integrates a Convolutional Autoencoder Neural Network and a Multi-Scale Convolutional Neural Network with deep layer aggregation into an ensemble learning architecture for robust TB detection from chest radiographs. The framework was evaluated on two public datasets and one private dataset, achieving 99% sensitivity and 94% specificity on the Shenzhen dataset, and consistently high accuracy across all datasets. Expert radiologists reviewed a subset of the predictions, confirming the clinical relevance and diagnostic reliability of the model. The ensemble approach demonstrated strong generalisability, effectively identifying active pulmonary TB in chest X-rays from a globally representative cohort. It also outperformed existing classifiers, achieving a state-of-the-art Area Under the Receiver Operating Characteristic of 0.98. These results highlight the potential of our approach as a practical and scalable tool for TB screening, particularly in low- and middle-income countries where radiological resources are limited.

## Introduction

Tuberculosis (TB) has been treated effectively through advancements in medical science since the mid-19th century. However, TB remains a major global health issue, with 10.8 million people contracting the disease in 2023 and 1.25 million deaths in 2023, according to the latest report of the World Health Organisation (WHO)^1^. TB continues to be one of the top infectious disease killers worldwide, particularly affecting low- and middle-income countries. The global incidence rate has declined 8.3% between 2015 and 2023, reflecting progress in TB control efforts. However, the burden remains high in many regions, especially in South-East Asia, which accounts for nearly half of all global TB cases. Despite improvements, Thailand remains listed among the 30 high TB burden countries. This persistent burden underscores the need for continued investment in TB control strategies. One of the key challenges in Thailand is the diagnostic gap, especially in high-volume healthcare settings. At Songklanagarind Hospital , Southern Thailand’s leading university hospital, TB diagnoses account for only 59% of the expected cases, indicating a significant number of undetected or misdiagnosed patients. Chest X-ray (CXR) imaging plays a central role in nationwide screening programmes, as recommended by the WHO, and this hospital alone processes approximately about 80,000–90,000 per year, highlighting the immense scale of screening and diagnostic demands placed on the healthcare system.

CXR imaging is essential for early detection of active TB, especially in resource-limited areas where access to computed tomography (CT) may not be as accessible^2^. In Thailand, TB screening is conducted nationwide using CXR following WHO guidelines^3^. However, the vast number of CXR images collected annually at Songklanagarind Hospital has exceeded the interpretive capacity of radiologists, leading to a reliance on general practitioners and increasing the risk of misdiagnosis. This situation highlights the urgent need for automated and scalable diagnostic tools to support clinical decision-making enabling timely and accurate diagnoses while reducing radiologists’ workload.

Artificial Intelligence (AI), and in particular Deep Learning (DL), has demonstrated strong performance in medical image analysis, including TB detection and diagnosis. A robust AI solution for automated chest X-ray (CXR) analysis could therefore offer a cost-effective and scalable approach to TB screening, especially in under-resourced healthcare environments.

While recent DL models have achieved impressive results across various medical imaging tasks, many of these architectures are resource-intensive and require large, well-annotated training datasets. This poses a challenge for deployment in real-world clinical settings, particularly in low- and middle-income countries, where data availability and computational infrastructure may be limited. In such contexts, the practical feasibility of DL models depends not only on their diagnostic accuracy but also on their ability to generalise across diverse populations and imaging conditions. Recent developments in DL have demonstrated dramatically higher performance on a variety of medical imaging tasks including TB detection and diagnostics.

To address these challenges, ensemble learning has emerged as a promising strategy. By aggregating the outputs of multiple models, ensemble approaches can improve prediction robustness, reduce variance and bias, and enhance generalisability, especially when training data is limited or heterogeneous^4^. Our proposed framework leverages this principle by combining a Convolutional Autoencoder (CAE) and a multi-scale residual neural network (ResNet), aiming to deliver reliable TB classification performance across varied datasets without relying on computationally intensive architectures.

In this study, we make the following key contributions:

- We propose a novel end-to-end ensemble DL framework for automated TB diagnosis that integrates two independent models: a CAE and a multi-scale ResNet with deep layer aggregation. This hybrid approach combines CAE’s unsupervised feature extraction and multi-scale network’s robust feature representation to enhance diagnostic accuracy for TB detection from CXRs.
- We validate our model on three diverse datasets, including two publicly available datasets from the National Library of Medicine at the National Institutes of Health (NLM/NIH) (Montgomery County and Shenzhen) and a private clinical dataset from Songklanagarind Hospital in Thailand. This enables a robust evaluation across different populations and imaging conditions.
- We demonstrate that our ensemble model outperforms individual models, achieving state-of-the-art performance with AUROC values up to 0.99, and showing strong generalisability and clinical relevance.
- We conduct an in-depth analysis of model predictions with expert radiologists, who reviewed misclassified cases and provided clinical interpretations. This qualitative evaluation helped identify patterns in false positives and negatives, reinforcing the diagnostic reliability and practical utility of the proposed system.

The remainder of this paper is structured as follows. The *Related Work* section reviews prior research in the field. The *Materials and Methods* section details the data sources and the proposed methodology. The *Evaluation Framework* section describes the datasets and metrics used to assess performance. The *Results* section presents the experimental outcomes, while the *Discussion* section analyses these findings in depth. Finally, the *Conclusions* section summarises the main contributions and outlines future directions.

## Related work

DL models used high-level complex architectures^5^ to solve the medical image classification problems. In particular, artificial neural networks (ANNs) have been often used as a robust tool in radiology diagnostic for image classification^6^. Convolutional neural networks (CNN) is also used as a primary method for image classification^7^.

Hwang et al.^8^, and Lakhani and Sundaram^9^ are one of the first studies that use DL to classify TB. Lakhani and Sundaram^9^ presented deep convolutional neural networks (DCNNs) and obtained their best result using an ensemble of the AlexNet and GoogLeNet DCNNs. Liu et al.^10^ presented a method using CNN for TB detection in a large imbalanced and less-category dataset to classify TB cases. Yadav et al.^11^ also proposed a TB screening system with the FastAI tool that provides a quick modification and mixed and matched low-level components to create an approach for CXR image. The authors employed the ResNet-50 model^12^. Li et al.^13^ proposed a CNN model using feature extraction of Conv and the unsupervised features of autoencoder (AE) as AE-CNN block to detect abnormalities in the classification of TB using the whole region of interest (ROI) images. Norval et al.^14^ investigated TB detection from CXR images using a hybrid approach. The proposed method combined the original statistical computer-aided detection and CNN, which included image pre-processing and segmentation techniques. This hybrid approach helped to improve the contrast of the images.

In 2020, Rahman et al.^15^ used a transfer learning technique on 9 different CNN models (ResNet18, ResNet50, ResNet101, ChexNet, InceptionV3, Vgg19, DenseNet201, SqueezeNet, and MobileNet) for TB and non-TB normal cases classification from CXR images on an NLM/NIH Shenzhen dataset, Belarus dataset, NIAID TB dataset and RSNA dataset^16^. As a result, the classification accuracy has been improved in the image segmentation dataset. Furthermore, in 2021, Rahman et al.^17^ investigated the method of detecting TB from CXR images on the NLM/NIH Shenzhen dataset. Their model used three pre-trained neural networks, ResNet101, VGG19, and DenseNet201, along with extreme gradient boosting.

A significant number of the research make use of pre-trained standard architectures. Resnet^12^, DenseNet^18^, Inception^19^, VGG^20^, or AlexNet are examples of these designs^21^. The majority of these research do not provide methodological originality but rather report or compare the performance of several architectures on a specific task.

Some studies provide methodological originality by employing strategies that have been shown to increase model performance in other domains. For example, ensemble learning is another method to retrieve a better predictive result by combining the prediction from different classification models into a new robust classifier model^22^. Ayaz et al.^23^, proposed a novel TB detection technique that combines hand-crafted features with CNN models through ensemble learning. Similarly, Lakhani et al.^9^ proposed the detection of TB by using two CNN models to classify pulmonary TB and normal CXR images with an ensemble technique added to the classification model to improve the efficiency of the classifier. A modality-specific ensemble DL model proposed by Rajaraman and Antani^24^ has enhanced the generalisation performance using pre-trained customised CNN model and modality-specific features. Recently, Wang et al.^25^ introduced a DL model that employs the Pseudo Zernike moment (PZM) as the feature extractor and the deep stacked sparse AE (DSSAE) as the classifier.

**Fig. 1:**
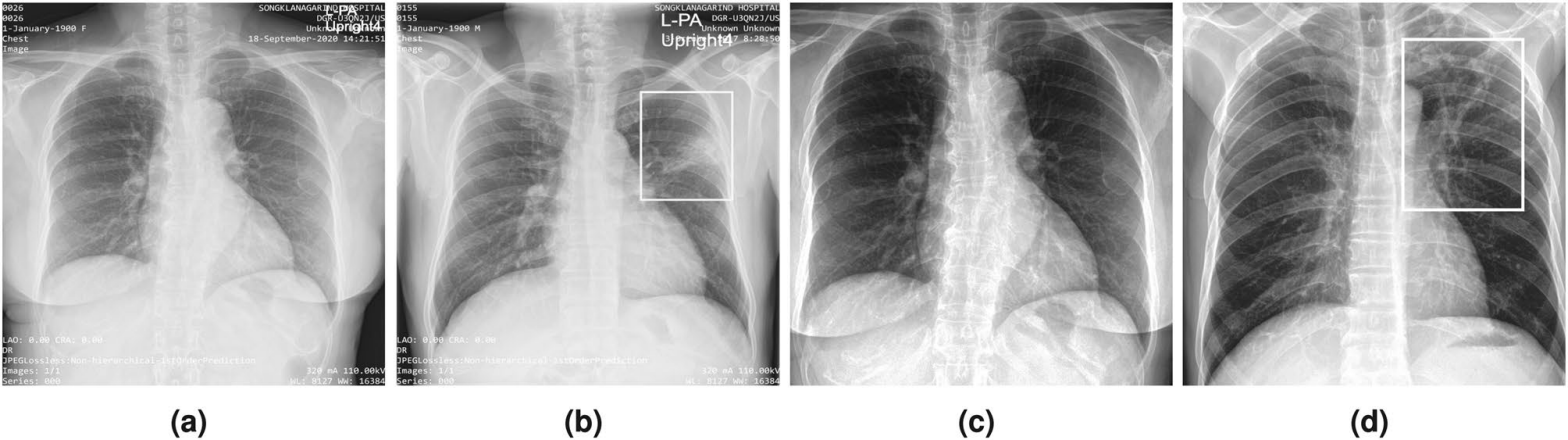
Example of chest X-rays (CXR). (**a**) Original CXR of a normal case; (**b**) Original CXR with active pulmonary tuberculosis. The white rectangle denotes reticulonodular infiltration; (**c**) Pre-processed CXR with normal findings; (**d**) Pre-processed CXR with active pulmonary tuberculosis. The white rectangle denotes reticulonodular infiltration.

Recently, deep learning approaches using Transformer architectures, such as Vision Transformers (ViTs), have shown promising performance in TB detection from CXRs. However, these models often come with limitations including high computational demands, restricted interpretability, and the need for extensive training datasets to generalise effectively across diverse clinical scenarios^26–28^.

## Materials and methods

This section presents the preprocessing steps applied to the raw CXR images, followed by a description of the proposed neural network (NN) models for TB diagnosis. It present two individual DL models, each designed to address TB diagnosis independently, as well as a novel ensemble method that combines their strengths. The first model is based on a convolutional AE (CAE) architecture, focusing on feature extraction through unsupervised learning. The second model employs a multi-scale ResNet network with deep layer aggregation to capture complex patterns at various scales. Finally, we detail an ensemble DL model that fuses the outputs of the CAE and the multi-scale ResNet, enhancing diagnostic accuracy through complementary information integration.

### Data pre-processing

This subsection focuses on the data pre-processing steps designed to enhance the quality of CXR images for TB diagnosis. The primary goal of pre-processing is to improve image visibility for key objects, such as nodules and fibrotic scars, significantly enhancing the performance of downstream processing tasks^29,30^.

Initially, all CXR scans in Digital Imaging and Communications in Medicine (DICOM) format were loaded using the VOI LUT provided in the DICOM header, ensuring appropriate windowing based on modality-specific metadata. To enhance contrast in diagnostically relevant regions, the pixel intensity values were normalised by setting the 0.5th percentile as the minimum and the 99.5th percentile as the maximum. All images were then converted to 8-bit grayscale (0–255 range) PNG files using the dicom2png utility, facilitating compatibility with standard image processing tools. Finally, histogram equalisation was applied to improve contrast within the anatomic region of interest in each image. This technique, commonly used for enhancing visibility in low-contrast areas, redistributes intensity levels to better spread out the most frequent pixel values, thereby improving contrast^31^.

Given a gray level *i* with $n_{i}$ occurrences, the probability of an occurrence of a pixel at level *i* in the image is:

1 $$\begin{aligned} p_{x}(i)=p(x=i)={\frac{n_{i}}{n}},\quad 0\le i<L \end{aligned}$$

where *L* is the number of possible intensity values (typically 256), *n* is the total number of pixels in the image, and $p_{x}(i)$ is the image’s histogram for pixel value *i*, normalised to [0, 1]. Let *p* represent a normalised histogram of *f* with a bin for each intensity level. The histogram equalised image *g* will be given as:

2 $$\begin{aligned} g_{i,j}=\lfloor {\{(L-1)\sum _{n=0}^{f_{i,j}}p_{n}}\rfloor \end{aligned}$$

where $\lfloor \cdot \rfloor$ returns the closest integer. The images were scaled to a [0, 1] pixel value range, and histogram equalisation was performed.

An additional automated central cropping step was applied to isolate lung fields and remove embedded markings, as recommended for CXR preprocessing. This lung field cropping not only reduces data loss from downscaling but also normalises geometric variations across images. Due to GPU memory constraints, the images were downsampled to $512 \times 512$ pixels.

For both NLM/NIH collections, images initially contained large black margins around the borders. These margins were removed, followed by histogram equalisation, and then central cropping was applied again to ensure the lung field was appropriately centered and the embedded markings on the CXR were removed. Figure 1 displays examples of original CXR images and the corresponding pre-processed versions.

### Convolutional autoencoder based DL (CAE-NN)

An AE is a type of neural network that learns to replicate its input as the output, typically without requiring labeled data, making it an unsupervised learning technique. In essence, an AE is trained for replicating the input to the output. There is a hidden layer that provides a ‘compressed’ code that lies in a space called latent space for representing the input. Briefly, an AE is composed of two major components: an encoder that converts the input into code and a decoder that converts the code into a reconstruction of the input. The dimensionality of AEs’ latent spaces is smaller than that of the original input, implying that its code cannot store a complete duplicate of the input data and requiring the model to learn efficient, lower-dimensional representations, how to represent the same data while preserving its most relevant features.

The CAE-NN leverages convolutional layers within the encoder and decoder to capture spatial patterns within the CXR images, which is particularly valuable for medical imaging tasks where structural details are critical. This architecture enables the CAE-NN to learn compact, high-level representations of the CXRs, contributing to the overall robustness and performance of the TB diagnostic process.

*Latent representation extraction* The primary hyperparameters in the convolutional autoencoder (CAE) architecture include the number of convolutional layers, the number of filters, the convolutional kernel size, and the number of strides^32^. In this work, these hyperparameters were manually tuned to optimise model performance. The resulting architecture of the CAE, as shown in Table 1, illustrates the details of each layer configuration.

**Table 1:** Detailed architecture of the proposed convolutional autoencoder including the number of channels and kernel size in each layer.

Encoder					Decoder				
Layer	Channels	Kernel	Strides	Output	Layer	Channels	Kernel	Strides	Output
INPUT	1	–	–	$512 \times 512$	CONVT1	128	$3 \times 3$	2	$8 \times 8$
CONV1	32	$3 \times 3$	2	$256 \times 256$	CONVT2	128	$3 \times 3$	2	$16 \times 16$
CONV2	32	$3 \times 3$	2	$128 \times 128$	CONVT3	64	$3 \times 3$	2	$32 \times 32$
CONV3	64	$3 \times 3$	2	$64 \times 64$	CONVT4	64	$3 \times 3$	2	$64 \times 64$
CONV4	64	$3 \times 3$	2	$32 \times 32$	CONVT5	32	$3 \times 3$	2	$128\times 128$
CONV5	128	$3 \times 3$	2	$16 \times 16$	CONVT6	32	$3 \times 3$	2	$256 \times 256$
CONV6	128	$3 \times 3$	2	$8 \times 8$	OUTPUT	1	–	–	$512 \times 512$

Each convolution (CONV) or transposed convolution (CONVT) layer is followed by an instance normalisation layer and a parameterised rectified linear unit (PReLU) layer.

The encoder in this CAE model comprises six convolutional blocks, each with a convolution layer (with a kernel size of $3 \times 3$ and the strides of 2 to half the size of features), an instance normalisation layer, and a parametric rectified linear unit (PReLU). PReLU is an activation function that generalises the classic rectified unit by adding a slope for negative values^33^. This arrangement allows the embedding of a large CXR image into the latent vector. Inversely, the decoder in our model consists of six transposed convolution blocks, each with a transposed convolution layer (with a kernel size of $3 \times 3$ and the strides of 2 to double the size of features), an instance normalisation layer, and a PReLU activation. The decoder reconstructs the CXR image from the latent vector. Each convolution layer is followed by a PReLU activation, allowing the encoding and decoding functions to be non-linear. Throughout the training phase, no label information is used, making this an entirely unsupervised approach. This unsupervised training enables the CAE to focus purely on learning patterns within the data itself, helping capture complex structures in the CXR images that are critical for subsequent TB diagnostic tasks.

**Fig. 2:**
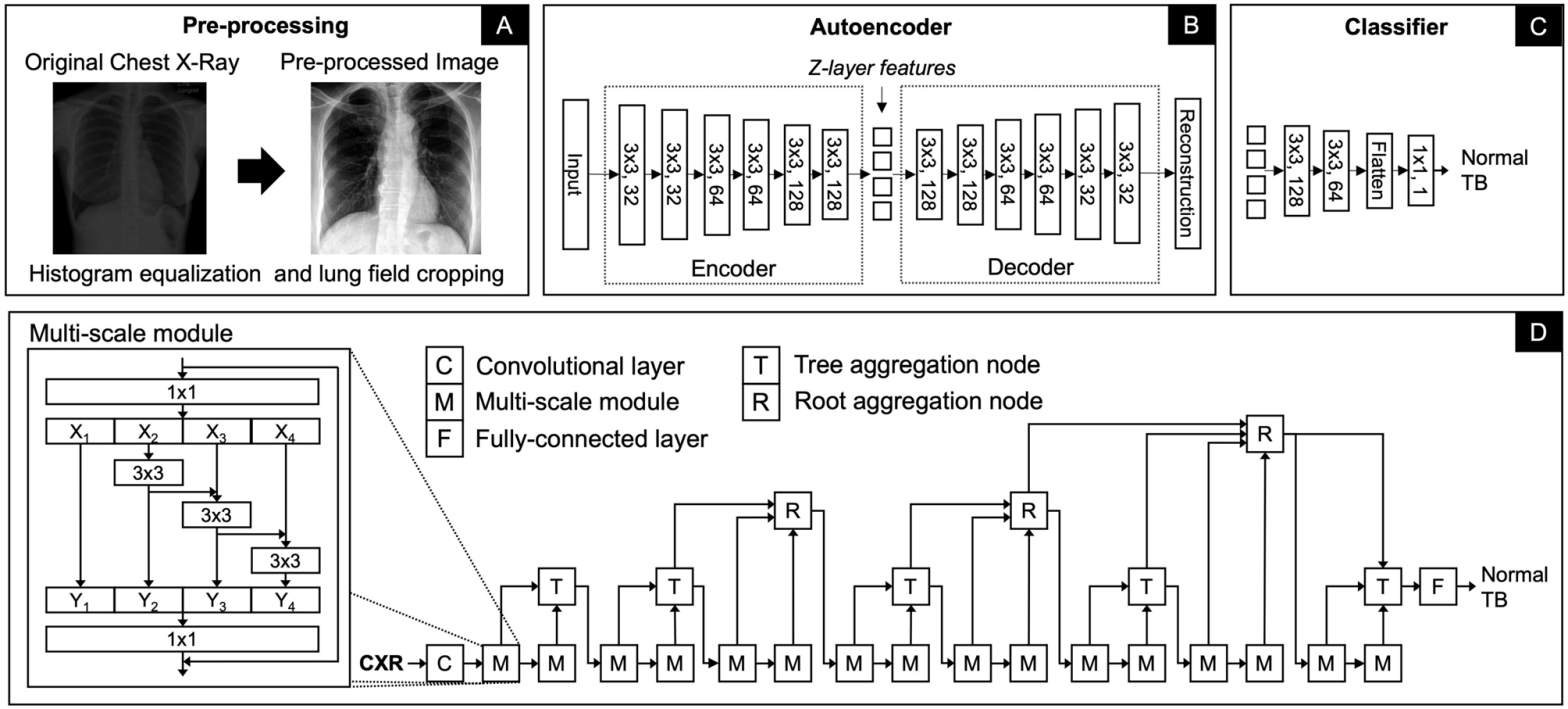
Overview of the proposed architectures used for feature extraction and classification for automated pulmonary tuberculosis detection. (**a**) Pre-processing: Chest X-rays undergo enhancement with histogram equalisation and lung field cropping to improve image quality; (**b**) An autoencoder is trained on chest X-ray images, with latent variables from the bottleneck layer for subsequent classification; (**c**) Classifier: classification is performed; (**d**) Multiscale Convolutional Neural Network: End-to-end classification is conducted, with features extracted from the last convolutional layer for ensemble learning.

*Classification* The classifier is designed to use latent features extracted by the deep CAE network. After the encoder’s final convolutional layer, the resulting two-dimensional feature matrix is processed through two additional $3 \times 3$ convolutional layers, configured with 256 and 128 output nodes, respectively. Each convolutional layer is followed by an instance normalisation and a PReLU activation. The resulting features are then flattened into one-dimensional vector and then fed to a dense layer with one output. The sigmoid activation function was used in the output layer. The training process is divided into two stages: in the first stage, the weights of the pre-trained autoencoder layers are frozen, and only the classifier layers are trained. This approach enables the classifier to learn without affecting the learned feature representations In the second stage, all of the layers are fine-tuned. An overview of this proposed method is illustrated in Fig. 2.

### Multi-scale convolutional neural network (MS-CNN)

A multi-scale ResNet^34^ with deep layer aggregation^35^ is proposed for improved feature extraction and classification. ResNets are typically composed of sequentially stacked residual blocks, each containing convolutional layers with non-linearity and shortcut connections. In this work, the original ResNet^36^ is extended with multi-scale backbone modules and hierarchical layer connections. The reason for this network’s selection is that MS-CNN not only results in enhanced feature representation but is also computationally efficient.

ResNet residual block with grouped convolution^36^ and multi-scale feature representation^34^ is employed in this work (see Fig. 2) following the implementation of Gao et al.^34^. The proposed residual block is made of 1) a convolutional layer with a kernel size of 1, 2) a hierarchical multi-scale module of three convolutional layers with a kernel size of 3 and a cardinality of 8, and 3) a convolutional layer with a kernel size of 1. In the multi-scale module, the input is split into four groups of equal sizes. Each subset is processed through a $3 \times 3$ convolutional layer, except the first group. The output from the first convolutional layer is also added to the input of the second convolutional layer. Similarly, the output from the second convolutional layer is added to the input of the third convolutional layer. Each pass to the convolutional layer enlarges a receptive field. All resulting feature maps are then concatenated together. Batch normalisation was applied after each convolutional layer in the multi-scale module. This creates the combinatorial explosion effect such that the output of the residual block has feature maps with different receptive field sizes^34^.

Multiple multi-scale residual blocks are combined in a hierarchical tree structure through the deep layer aggregation scheme similar to the work by Yu et al.^35^ (see Fig. 2). The hierarchical aggregation uses iterative connections joining neighbouring residual blocks into a tree and hierarchical connections joining multiple trees, facilitating feature map propagation and gradients across the network. With multi-scale residual blocks, the hierarchical tree structure can promote even stronger multi-scale feature representation to the network.

The proposed model comprises a convolutional layer with a kernel size of 7 with a stride of 2, followed by 16 multi-scale residual blocks arranged in the deep layer aggregation structure. This architecture includes eight iterative aggregation nodes, of which five are tree aggregation nodes, and three are root aggregation nodes. Each aggregation node is composed of a convolutional layer with a kernel size of 1, batch normalisation, and a PReLU activation. Prior to each tree aggregation node, a convolutional layer with a kernel size of 7 with a stride of 2 is applied to half the size of feature maps. The multi-scale network has one output and a sigmoid activation function.

### Ensemble learning

Ensemble learning combines multiple base models to perform tasks such as supervised and unsupervised learning, often outperforming individual models. A key factor in the effectiveness of ensemble techniques is the diversity among the base classifiers, as highlighted by Dietterich^37^. In this study, two distinct deep DL models were employed to create a diverse ensemble classifier.

To implement this ensemble approach, we concatenated the two-dimensional feature maps from the last convolutional layer of the encoder in the CAE-NN model with those from the last convolutional layer of the MS-CNN model. Both sets of the feature maps have the same size of feature maps at a downsampling factor of $2^6$ of the input size, ensuring compatibility in dimensions. The concatenated feature maps are then passed through the same classifier architecture used in the CAE-NN model, enabling the ensemble model to classify CXR as either normal or indicative of TB. Importantly, no additional hyperparameter tuning was performed for the ensemble classifier; instead, we reused the configuration from the CAE-NN classifier to maintain consistency and avoid overfitting. This fusion approach leverages the strengths of both models to enhance diagnostic accuracy while preserving architectural simplicity.

## Evaluation framework

This section provides an overview of the datasets and metrics used to evaluate the performance of the proposed models.

### Datasets

The efficiency of the proposed methodology was evaluated using two public datasets from the NLM/NIH and a private dataset containing cases from Songklanagarind Hospital. Table 2 presents the demographic and diagnostic information for participants in each dataset.

**Table 2:** Patient demographics and diagnostic information of the study population. TB = Tuberculosis cases; HC = Healthy Controls.

Dataset	Number of cases		Gender (%)		Age (years)
	TB	HC	Male	Female	
Montgomery County, United States (MC)	58	80	46	54	40.1 ± 18.7
Shenzhen No.3 People’s Hospital, China (SZ)	336	326	69	31	35.6 ± 14.7
Songklanagarind Hospital, Thailand (SK)	268	274	50	50	51.2 ± 18.1

The table includes the number of TB and HC cases, gender distribution, and average age ($\pm ~$standard deviation) for each dataset.

#### NLM collection—Montgomery County X-ray dataset (MC)

The CXR images in this dataset were obtained through the TB control programme of Montgomery County’s Department of Health and Human Services in Montgomery County, Maryland, USA^16^. This collection comprises 138 posterior-anterior X-rays, 80 of which are normal and 58 of which are abnormal with TB symptoms. The CXRs were captured using a Eureka stationary X-ray machine (CR) and are supplied as 12-bit grey level images in Portable Network Graphics (PNG) format.

#### NLM collection—Shenzhen hospital X-ray dataset (SZ)

This CXR dataset was collected at Shenzhen No.3 Hospital in Shenzhen, Guangdong Province, China^16^. It contains 326 normal CXRs and 336 abnormal CXRs exhibiting different TB symptoms.

#### Songklanagarind hospital dataset (SK)

The Songklanagarind Hospital dataset was collected by the Department of Radiology, Faculty of Medicine, Prince of Songkla University, Thailand. The CXRs were obtained from patients aged 15 years old or over who had undergone both CXR and chest Computed Tomography (CT) between November 2015 and December 2020. Full-text radiologist reports accompany the CXRs.

Patients with Human Immunodeficiency Virus (HIV) positive serology were excluded from the dataset. This decision was based on clinical grounds: TB in HIV-positive individuals often presents with atypical or non-specific radiographic features, which differ substantially from those observed in immunocompetent patients. Including such cases would have introduced significant heterogeneity into the dataset, potentially compromising the model’s ability to learn consistent diagnostic patterns. As our model was specifically designed to detect active pulmonary TB in the general population, we opted to exclude these cases to maintain clinical and methodological consistency. Additionally, CXRs lacking posteroanterior or anteroposterior positioning, those of poor image quality, and cases with inconsistent dates between diagnosis history, CXR, and CT were removed from the dataset. We also excluded patients who had inconsistent dates for diagnosis history, CXR, and CT. All methods were carried out in accordance with relevant guidelines and regulations, including the Declaration of Helsinki. The study protocol was reviewed and approved by the Human Research Ethics Committee (HREC) of the Faculty of Medicine, Prince of Songkla University (approval No. REC.61-424-9-1). The requirement for informed consent was waived by the HREC of the Faculty of Medicine, Prince of Songkla University, as the study involved a retrospective review of anonymised data.

Importantly, this dataset presents a significantly higher level of diagnostic complexity compared to commonly used public datasets. It encompasses a broad spectrum of abnormalities–including small and large opacities, masses, cavities, fibrosis, calcifications, and pleural effusions–that pose challenges even for experienced radiologists. Table 3 summarises the prevalence of key lesion types in normal and TB cases.

**Table 3:** Lesion prevalence in normal and tuberculosis chest X-rays in the Songklanagarind hospital dataset.

Lesion	Normal	Tuberculosis
Small Opacity	27	208
Large Opacity	2	202
Mass/Nodule	0	68
Cavity	0	131
Fibrosis	6	105
Calcification	3	36

Images may contain multiple lesion types.

Each CXR, whether normal or showing active pulmonary TB, was reviewed and interpreted by experienced thoracic radiologists. Active TB cases were confirmed by sputum culture for Mycobacterium TB. The CXRs were exported in uncompressed DICOM format from the Picture Archiving and Communication System (PACS), resulting in a final dataset of 542 CXRs–274 normal images and 268 with confirmed active pulmonary TB.

### Evaluation strategy

AEs consist of an encoder and a decoder, trained concurrently to minimise a loss function between an input and the reconstruction of the input. Commonly used loss functions for AE training include mean-squared error (MSE) and binary cross-entropy (BCE). In this work, MSE is used to evaluate the reconstruction quality of the AE on test images, assessing how accurately the AE can recreate previously unseen images. When evaluating the reconstructed outcomes, a quality metric is essential for comparing the original and reconstructed images. In this study, the peak signal-to-noise ratio (PSNR) was used as a quality measurement. PSNR provides a quantitative assessment of the reconstruction’s fidelity by comparing the maximum possible power of a signal (in this case, the original image) to the power of corrupting noise that affects the fidelity of its representation (the reconstructed image).

As the PSNR increases, the quality of the reconstructed image improves. In our experiments, we achieved an average PSNR values of 30.86 dB, 31.71 dB, and 35.51 dB for the reconstructed validation images from the Montgomery County, the Shenzhen Hospital, and the Songklanagarind Hospital datasets, respectively.

The proposed models predict whether a CXR belongs to the normal or the TB class. To evaluate the classification performance of the proposed methods across the three datasets, four key metrics are used: accuracy, sensitivity, specificity, and F1-score. These measures provide a comprehensive evaluation of the classification performance of the proposed models, allowing for insights into their effectiveness across different datasets.

Accuracy measures the proportion of correctly classified instances (both normal and TB) out of the total instances:

3 $$\begin{aligned} \text {Accuracy} = \frac{\text {TP}+\text {TN}}{\text {TP}+\text {TN}+\text {FP}+\text {FN}}. \end{aligned}$$

Sensitivity (Recall) assesses the ability of the model to correctly identify a chest radiograph as pulmonary TB:

4 $$\begin{aligned} \text {Sensitivity} = \frac{\text {TP}}{\text {TP}+\text {FN}}. \end{aligned}$$

Specificity measures the model’s ability to correctly classify a CXR as normal findings:

5 $$\begin{aligned} \text {Specificity} = \frac{\text {TN}}{\text {TN}+\text {FP}}. \end{aligned}$$

$\hbox {F}_1$-Score is the harmonic mean of precision and recall, providing a balance between the two metrics. It is particularly useful in scenarios where class distributions are imbalanced, as it accounts for both false positives and false negatives:

6 $$\begin{aligned} \text {F}_1\text {-Score} = \frac{\text {TP}}{\text {TP}+\frac{1}{2}(\text {FP}+\text {FN})} \end{aligned}$$

The 5-fold cross-validation approach is employed to build a robust model. This method is useful for dealing with the overfitting problem.

Data augmentation is employed to generate additional labeled data for training the models in this work. Specifically, random affine transformations are performed with a rotation range between $-\frac{\pi }{8}$ and $-\frac{\pi }{8}$, a scale range between 0.80 and 1.20, and a translation range of -64 to 64 pixels. Additionally, random elastic transformations are applied with a grid spacing of 64 pixels and a magnitude between 0 and 2. This augmentation is applied on-the-fly during training to enhance the diversity of the training dataset. To prevent data leakage, the data is initially separated, ensuring that augmentation is conducted exclusively on the training set for each training cycle^38^.

All networks were implemented using the Project MONAI framework^39^ (version 0.5) on an Nvidia GeForce RTX 2080 GPU. A cross-entropy loss function is used, and the networks are trained using the NovoGra method^40^ to optimise the training process.

## Results

This section details the results produced for TB classification using the techniques and evaluation framework presented in previous sections.

**Table 4:** Classification performance of the proposed models on three datasets without data augmentation.

Metrics	Dataset								
	Songklanagarind Hospital (SK)			Montgomery County (MC)			Shenzhen No.3 People’s Hospital (SZ)		
	MS-CNN	CAE-NN	Ensemble	MS-CNN	CAE-NN	Ensemble	MS-CNN	CAE-NN	Ensemble
AUROC	0.95 ± 0.02	0.93 ± 0.03	**0.96** ± **0.03**	0.76 ± 0.10	0.65 ± 0.14	**0.77** ± **0.09**	0.94 ± 0.02	0.90 ± 0.03	**0.98** ± **0.01**
Accuracy	**0.92** ± **0.03**	0.88 ± 0.04	0.92 ± 0.05	0.75 ± 0.03	0.71 ± 0.10	**0.77** ± **0.09**	0.89 ± 0.03	0.85 ± 0.03	**0.95** ± **0.04**
Sensitivity	0.86 ± 0.03	**0.91** ± **0.08**	0.89 ± 0.07	** 0.80** ± **0.15**	0.69 ± 0.17	0.73 ± 0.23	0.92 ± 0.05	0.87 ± 0.04	**0.95** ± **0.04**
Specificity	**0.96** ± **0.03**	0.85 ± 0.10	0.95 ± 0.05	0.69 ± 0.25	0.74 ± 0.16	**0.83** ± **0.14**	0.86 ± 0.02	0.83 ± 0.07	**0.95** ± **0.06**
PPV	**0.96** ± **0.03**	0.87 ± 0.07	0.95 ± 0.05	0.81 ± 0.11	0.79 ± 0.08	**0.88** ± **0.09**	0.86 ± 0.02	0.84 ± 0.06	**0.95** ± **0.06**
NPV	0.88 ± 0.03	** 0.91** ± **0.06**	0.90 ± 0.06	** 0.76** ± **0.14**	0.64 ± 0.12	0.73 ± 0.17	0.92 ± 0.05	0.87 ± 0.03	**0.95** ± **0.04**

MS-CNN stands for multi-scale convolutional neural network, whereas CAE-NN is short for convolutional autoencoder based classifier. The performance metrics used are area under the receiver operating characteristics (AUROC), accuracy, sensitivity, specificity, positive predictive value (PPV), and negative predictive value (NPV). **Bold** values indicate the best performance for each metric and dataset.

**Table 5:** Classification performance of the proposed models on three datasets with data augmentation.

Metrics	Dataset								
	Songklanagarind Hospital (SK)			Montgomery County (MC)			Shenzhen No.3 People’s Hospital (SZ)		
	MS-CNN	CAE-NN	Ensemble	MS-CNN	CAE-NN	Ensemble	MS-CNN	CAE-NN	Ensemble
AUROC	**0.97** ± **0.02**	0.94 ± 0.03	**0.97** ± **0.02**	0.74 ± 0.13	0.70 ± 0.13	**0.77** ± **0.06**	0.98 ± 0.01	0.95 ± 0.02	**0.99** ± **0.01**
Accuracy	**0.93** ± **0.03**	0.88 ± 0.04	**0.93** ± **0.02**	**0.77** ± **0.08**	0.72 ± 0.10	**0.77** ± **0.04**	0.94 ± 0.02	0.91 ± 0.02	**0.96** ± **0.02**
Sensitivity	0.91 ± 0.08	0.89 ± 0.06	**0.94** ± **0.06**	**0.86** ± **0.11**	0.80 ± 0.15	0.81 ± 0.12	0.95 ± 0.03	0.94 ± 0.03	**0.99** ± **0.01**
Specificity	**0.96** ± **0.03**	0.88 ± 0.08	0.92 ± 0.02	0.63 ± 0.15	0.62 ± 0.17	**0.73** ± **0.12**	**0.94** ± **0.05**	0.88 ± 0.07	**0.94** ± **0.04**
PPV	**0.96** ± **0.03**	0.89 ± 0.06	0.93 ± 0.02	0.77 ± 0.07	0.75 ± 0.08	**0.80** ± **0.06**	**0.94** ± **0.05**	0.89 ± 0.06	**0.94** ± **0.03**
NPV	0.92 ± 0.07	0.89 ± 0.05	** 0.94** ± **0.06**	**0.79** ± **0.16**	0.72 ± 0.13	0.78 ± 0.09	0.95 ± 0.03	0.94 ± 0.03	**0.99** ± **0.01**

MS-CNN stands for multi-scale convolutional neural network whereas CAE-NN is short for convolutional autoencoder based classifier. The performance metrics that are used in the experiments are : Area under the receiver operating characteristics (AUROC), accuracy, sensitivity, specificity, positive predictive value (PPV), and negative predictive value (NPV). **Bold** values indicate the best performance for each metric and dataset.

**Fig. 3:**
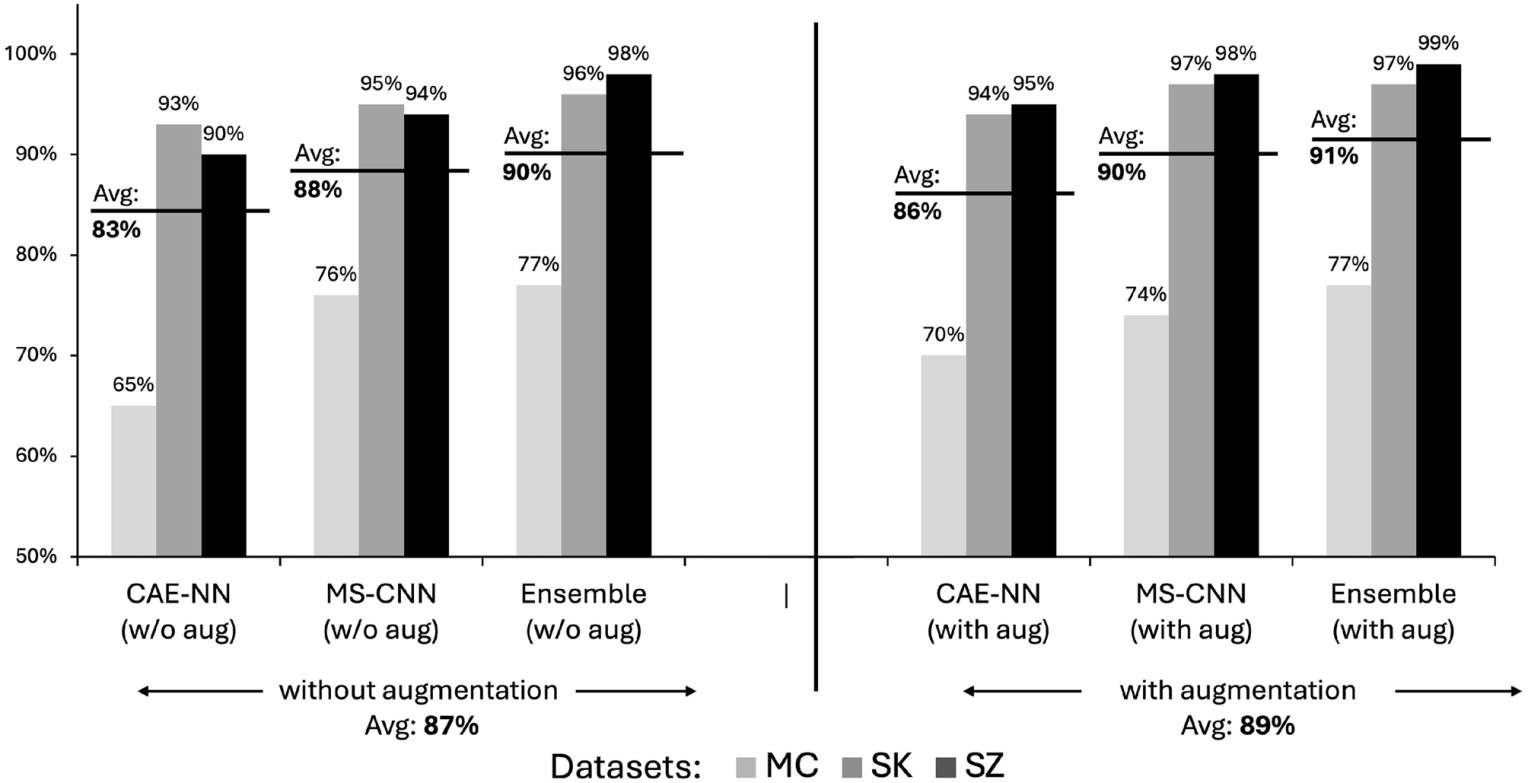
Area under the receiver operating characteristics (AUROC) comparison across classification models and datasets.

Tables 4 and 5 present the classification performances of our models for all datasets, both without and with data augmentation, respectively. Figure 3 shows the comparison of AUROC rates among three models (without and with data augmentation) across the SK, MC, and SZ datasets.

Data augmentation led to a slight performance increase in all datasets. Notably, MS-CNN consistently outperformed CAE-NN across all datasets in terms of AUROC. Furthermore, the ensemble of both methods yielded an additional performance boost. CAE-NN exhibited larger standard deviations compared to MS-CNN.The ensemble model achieved AUROC values of 0.97, 0.77, and 0.99 for the SK, MC, and SZ datasets, respectively. Additionally, the performance of the models for the MC dataset was lower compared to the other two datasets across nearly all metrics.

## Discussion

Three distinct datasets were used to validate the performance of our models, containing participants from three different countries (Thailand, USA, and China) which likely reflect a degree of demographic and ethnic diversity, although detailed patient-level demographic data were not available. They also represent a range of imaging protocols, clinical settings, and file formats (DICOM and PNG). It is important to note that the MC dataset is relatively small in size, which may have contributed to its lower performance metrics. To mitigate the effects of limited data, we employed extensive data augmentation and a 5-fold cross-validation strategy to enhance model robustness and reduce the risk of overfitting.

Despite the size of the datasets, in this binary classification scenario, all networks demonstrated remarkable performance in distinguishing between TB and normal images across the three datasets, indicating that the model can effectively generalise across diverse populations and healthcare settings. Thus, the proposed approach demonstrates high potential for generalisability to heterogeneous datasets.

Thoracic radiologists expert interpretations were not only used to establish SK dataset diagnosis but also played a critical role in the qualitative analysis of model performance, particularly in identifying and understanding misclassifications. Notably, the radiologists highlighted that the SK dataset differs slightly from the MC and SZ datasets, as it comprises only active TB cases, whereas the MC and SZ datasets include both active TB and old TB lesions. Despite these differences, the models achieved similar performance levels across different datasets, with the exception of the MC dataset. This discrepancy may be attributed to the smaller number of cases in the MC dataset compared to the others.

**Fig. 4:**
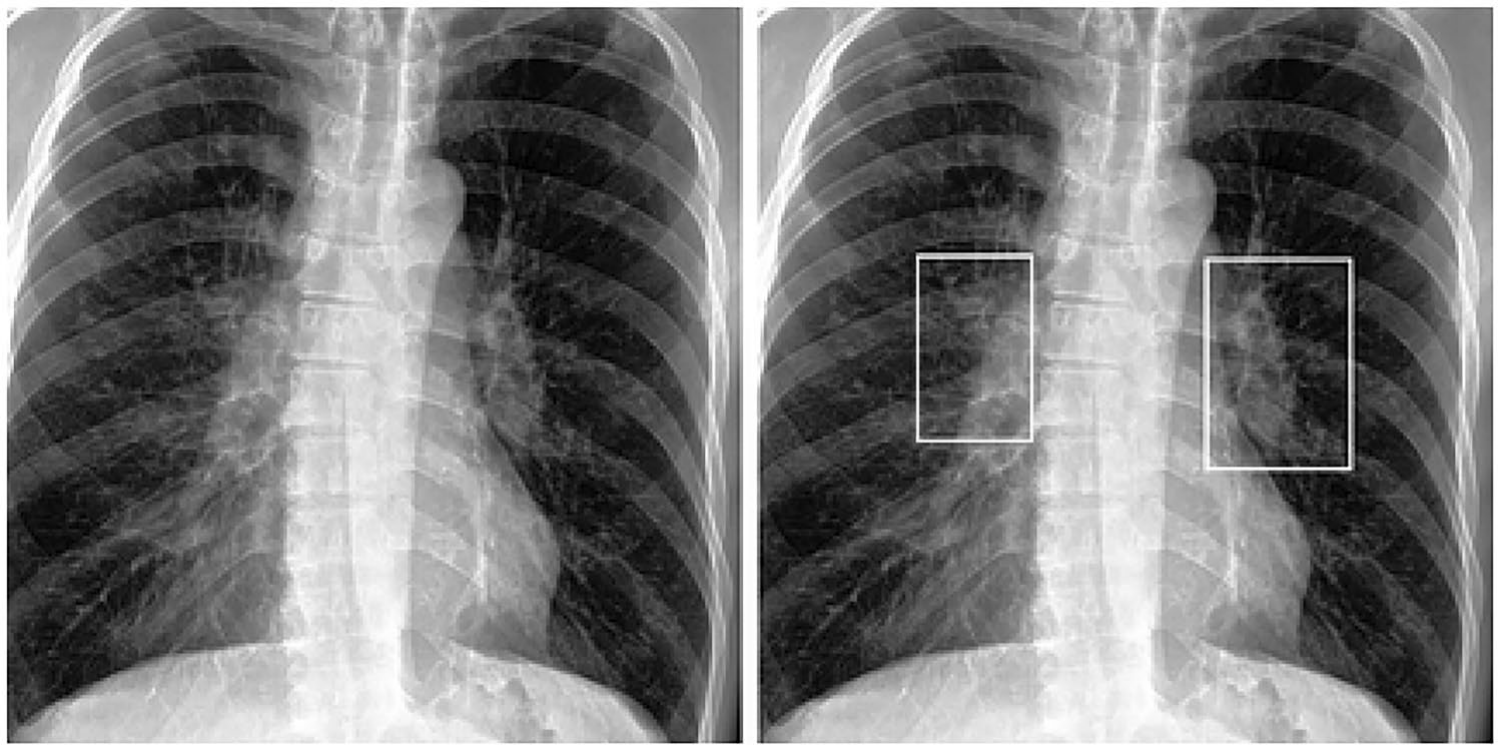
Example of miss-classified result as the false negative chest X-ray image from the single Deep Learning model (a patient with active pulmonary tuberculosis, positive, but classified as negative). The necrotic mediastinal nodes identified by an expert radiologist are denoted with the white rectangles.

The use of our institutional dataset (SK Dataset) allows us to extend the analysis to full-text radiologist reports, which provided critical clinical insight beyond initial labelling. When employing a single DL model (either MS-CNN or CAE-NN) on the SK dataset, we observed that most false-positive predictions corresponded to CXRs displaying some abnormalities without active TB. Examples of these abnormalities include prominent heart size, dilated aorta, suboptimal inspiration, degenerative spine, and slightly oblique positioning. Conversely, the majority of false-negative predictions were associated with CXRs that exhibited minimal abnormalities, indeterminate TB activity, or abnormalities localised in the upper lung zones. Figure 4 shows a sample of false-negative cases identified by a single DL model. The left image in the figure shows the original CXR, which neither the MS-CNN nor the CAE-NN models were able to identify as indicative of active TB. The right image presents the same CXR after further investigation, which included a patient’s sputum acid-fast stain test and an expert radiologist’s evaluation. The test results confirmed that the patient had active TB disease, and the expert radiologist identified an active TB lesion, specifically necrotic mediastinal nodes, on the CXR. An interesting finding from this investigation was that the lesion’s pattern in the image was atypical, as only the necrotic mediastinal nodes were visible, with no apparent infiltration. Furthermore, the lesion was obscured by a part of the rib bone, as shown in the figure. Nevertheless, when using the ensemble model, this image was correctly classified as a positive result.

In the MC dataset, as observed in its detailed findings, most false-negative predictions were associated with CXRs showing abnormalities in the upper lung zones and old TB scars. Similarly, on the SZ dataset, half of the false-negative predictions involved CXRs with upper lung zone abnormalities, while one-third were related to the reactivation of old TB lesions. Notably, most false positives and negatives arose from cases that were not prominently represented in the datasets. Further investigation into bone suppression algorithms may enhance classification accuracy for detecting TB in upper lung regions or oblique positions. It is important to note that the SK dataset comprises only CXRs with active TB, whereas the MC and SZ datasets include CXRs with both active TB and scars from previous infections.

Our results indicate that the proposed ensemble model significantly improved classification accuracy compared to the individual CAE-NN and MS-CNN models. The reported false-negative rates from the interpretation of expert radiologists were from 7% to 9%^41,42^. However, the interpretation of the general physician and other specialists had a false negative rate, ranging from 15% to 30%^43^. Thus, the ensemble DL could serve as a valuable triage tool for screening abnormal CXRs, particularly for annual health check-ups and preoperative assessments in developing countries where the availability of expert radiologists is limited. These tools have the potential to play a critical role in the diagnostic process, especially in settings that radiologists are limited and require low-cost solutions.

While our study focused on evaluating the performance of two custom-designed architectures and their ensemble, we acknowledge the importance of benchmarking against other state-of-the-art methods. In particular, recent ViT-based models have shown promising results in TB classification tasks. Although these models often require substantial computational resources, future work will explore comparisons with pre-trained ViT architectures to assess performance and efficiency trade-offs. This will help contextualise our ensemble model within the broader landscape of TB detection approaches.

## Conclusions

This work presents a novel ensemble method together with two individual DL-based classification approaches for the automatic diagnosis of TB from CXR. The performance of the DL models was evaluated on two publicly available datasets (SK and MC) as well as on one private dataset(SZ), demonstrating their effectiveness in diverse clinical scenarios. Both DL-driven techniques yielded accurate and reliable pulmonary TB prediction and classification results. The experimental findings indicate that the proposed ensemble learning method significantly outperforms the individual models and achieves state-of-the-art results in automated pulmonary TB diagnosis using CXRs. Specifically, the ensemble model attained AUROC rates of 0.97, 0.77, and 0.99 on the SK, MC, and SZ datasets, respectively.

Nevertheless, model’s performance could be further enhanced with an increase in the number of cases, as the appearances of CXRs can vary widely due to differences in patient anatomy and associated abnormalities. Most false positives and negatives were linked to cases that were underrepresented in the datasets. Future work will also explore the integration of bone suppression algorithms to enhance lesion visibility, particularly in the upper lung regions, where TB signs are often subtle or obscured.

A key strength of this study was the involvement of experienced thoracic radiologists, who not only curated the SK dataset but also conducted in-depth analyses of misclassified cases. Their clinical insights were instrumental in identifying atypical TB presentations and understanding the limitations of the models, thereby reinforcing the clinical relevance of the findings.

Importantly, HIV-positive cases were deliberately excluded due to clinical considerations. While this limits the immediate applicability of the model to this patient subgroup, it also presents a compelling avenue for future work: the development of tailored diagnostic models that address the distinct radiological features of HIV-associated TB.

Although a formal reader study comparing the model’s performance to human experts was beyond the scope of this work, we recognise its value and consider it an important direction for future research. Such a study would provide a more comprehensive benchmark for evaluating the model’s diagnostic utility in real-world clinical settings.

Future work will focus on integrating newer datasets and refining the ensemble method to enhance robustness across diverse imaging conditions, ensuring broader applicability in global healthcare systems. This model has the potential to serve as a valuable and rapid diagnostic tool, addressing the challenges associated with delayed diagnosis or misdiagnosis of TB and ultimately saving countless lives.

## Data Availability

This study used three datasets for model training and evaluation. The two publicly available TB CXR datasets from the NLM are accessible at https://lhncbc.nlm.nih.gov/LHC-downloads/downloads.html#tuberculosis-image-data-sets. The third dataset, the Songklanagarind Hospital Dataset, is private and not publicly available due to confidentiality restrictions. Researchers interested in accessing the Songklanagarind Hospital Dataset may contact our co-author at thammasin.i@psu.ac.th for potential data-sharing agreements, subject to institutional and ethical approvals.

## References

[1] (WHO), W. H. O. Global tuberculosis report 2024 (2024). Licence: CC BY-NC-SA 3.0 IGO.

[2] Nachiappan, A. C. et al. Pulmonary tuberculosis: Role of radiology in diagnosis and management. *Radiographics* **37**, 52–72 (2017). 28076011 10.1148/rg.2017160032

[3] (WHO), W. H. O. WHO consolidated guidelines on tuberculosis module 2: Screening – systematic screening for tuberculosis disease (2021). Accessed on 6th November 2024. 33822560

[4] Ju, C., Bibaut, A. l. & van der Laan, M. The relative performance of ensemble methods with deep convolutional neural networks for image classification. *J. Appl. Stat.* **45**, 2800–2818 (2018). 10.1080/02664763.2018.1441383 PMC6800663 31631918

[5] Guo, Y. et al. Deep learning for visual understanding: A review. *Neurocomputing* **187**, 27–48 (2016).

[6] Kwon, T. et al. Diagnostic performance of artificial intelligence model for pneumonia from chest radiography. *PLOS ONE* **16**, 1–13. 10.1371/journal.pone.0249399 (2021). 10.1371/journal.pone.0249399 PMC8049482 33857181

[7] Yan, Y., Yao, X.-J., Wang, S.-H. & Zhang, Y.-D. A survey of computer-aided tumor diagnosis based on convolutional neural network. *Biology* **10**, 1084 (2021). 34827077 10.3390/biology10111084 PMC8615026

[8] Hwang, S., Kim, H.-E., Jeong, J. & Kim, H.-J. A novel approach for tuberculosis screening based on deep convolutional neural networks. In *Medical imaging 2016: computer-aided diagnosis*, vol. 9785, 750–757 (SPIE, 2016).

[9] Lakhani, P. & Sundaram, B. Deep learning at chest radiography: Automated classification of pulmonary tuberculosis by using convolutional neural networks. *Radiology* **284**, 574–582 (2017). 28436741 10.1148/radiol.2017162326

[10] Liu, C. et al. TX-CNN: Detecting tuberculosis in chest X-ray images using convolutional neural network. In *2017 IEEE international conference on image processing (ICIP)*, 2314–2318 (IEEE, 2017).

[11] Yadav, O., Passi, K. & Jain, C. K. Using deep learning to classify X-ray images of potential tuberculosis patients. In *IEEE International Conference on Bioinformatics and Biomedicine (BIBM)*, 2368–2375 (IEEE, 2018).

[12] He, K., Zhang, X., Ren, S. & Sun, J. Deep residual learning for image recognition. In *Proceedings of the IEEE Conference on Computer Vision and Pattern Recognition*, 770–778 (2016).

[13] Li, L., Huang, H. & Jin, X. AE-CNN classification of pulmonary tuberculosis based on ct images. In *2018 9th International Conference on Information Technology in Medicine and Education (ITME)*, 39–42 (IEEE, 2018).

[14] Norval, M., Wang, Z. & Sun, Y. Pulmonary tuberculosis detection using deep learning convolutional neural networks. In *Proceedings of the 3rd International Conference on Video and Image Processing*, 47–51 (2019).

[15] Rahman, T. et al. Reliable tuberculosis detection using chest X-ray with deep learning, segmentation and visualization. *IEEE Access* **8**, 191586–191601 (2020).

[16] Jaeger, S. et al. Two public chest X-ray datasets for computer-aided screening of pulmonary diseases. *Quant. Imag. Med. Surg.* **4**, 475 (2014). 10.3978/j.issn.2223-4292.2014.11.20 PMC4256233 25525580

[17] Rahman, M., Cao, Y., Sun, X., Li, B. & Hao, Y. Deep pre-trained networks as a feature extractor with XGBoost to detect tuberculosis from chest X-ray. *Comput. Electr. Eng.* **93**, 107252 (2021).

[18] Huang, G., Liu, Z., Van Der Maaten, L. & Weinberger, K. Q. Densely connected convolutional networks. In *Proceedings of the IEEE conference on computer vision and pattern recognition*, 4700–4708 (2017).

[19] Szegedy, C. et al. Going deeper with convolutions. In *Proceedings of the IEEE conference on computer vision and pattern recognition*, 1–9 (2015).

[20] Simonyan, K. & Zisserman, A. Very deep convolutional networks for large-scale image recognition. In *International Conference on Learning Representations (ICLR)* (2015).

[21] Krizhevsky, A., Sutskever, I. & Hinton, G. E. Imagenet classification with deep convolutional neural networks. *Adv. Neural Inf. Process. Syst.* **25** (2012).

[22] Fraz, M. M. et al. An ensemble classification-based approach applied to retinal blood vessel segmentation. *IEEE Trans. Biomed. Eng.* **59**, 2538–2548 (2012). 22736688 10.1109/TBME.2012.2205687

[23] Ayaz, M., Shaukat, F. & Raja, G. Ensemble learning based automatic detection of tuberculosis in chest X-ray images using hybrid feature descriptors. *Phys. Eng. Sci. Med.* **44**, 183–194 (2021). 33459996 10.1007/s13246-020-00966-0 PMC7812355

[24] Rajaraman, S. & Antani, S. K. Modality-specific deep learning model ensembles toward improving tb detection in chest radiographs. *IEEE Access* **8**, 27318–27326 (2020). 32257736 10.1109/access.2020.2971257 PMC7120763

[25] Wang, S.-H., Satapathy, S. C., Zhou, Q., Zhang, X. & Zhang, Y.-D. Secondary pulmonary tuberculosis identification via Pseudo-Zernike moment and deep stacked sparse autoencoder. *J. Grid Comput.* **20**, 1–16 (2022). 34931118 10.1007/s10723-021-09596-6 PMC8674408

[26] El-Ghany, S. A., Elmogy, M., A. Mahmood, M. & Abd El-Aziz, A. A. A robust tuberculosis diagnosis using chest X-rays based on a hybrid vision transformer and principal component analysis. *Diagnostics* **14**, 10.3390/diagnostics14232736 (2024). 10.3390/diagnostics14232736 PMC11640000 39682642

[27] Vanitha, K., Mahesh, T., Kumar, V. V. & Guluwadi, S. Enhanced tuberculosis detection using vision transformers and explainable AI with a Grad-CAM approach on chest X-rays. *BMC Med. Imag.* **25**, 96. 10.1186/s12880-025-01630-3 (2025). 10.1186/s12880-025-01630-3 PMC11934573 40128729

[28] Devasia, J., Goswami, H., Lakshminarayanan, S., Rajaram, M. & Adithan, S. Deep learning classification of active tuberculosis lung zones wise manifestations using chest X-rays: A multi label approach. *Sci. Rep.* **13**, 887. 10.1038/s41598-023-28079-0 (2023). 36650270 10.1038/s41598-023-28079-0 PMC9845381

[29] Misra, P. & Yadav, A. S. Impact of preprocessing methods on healthcare predictions. In *Proceedings of 2nd International Conference on Advanced Computing and Software Engineering (ICACSE)* (2019).

[30] Almuhaideb, S. & Menai, M. E. B. Impact of preprocessing on medical data classification. *Front. Comput. Sci.* **10**, 1082–1102 (2016).

[31] Sherrier, R. H. & Johnson, G. Regionally adaptive histogram equalization of the chest. *IEEE Trans. Med. Imag.* **6**, 1–7 (1987). 10.1109/TMI.1987.4307791 18230420

[32] Yagis, E., García Seco de Herrera, A. & Citi, L. Convolutional autoencoder based deep learning approach for alzheimer’s disease diagnosis using brain mri. In *2021 IEEE 34th International Symposium on Computer-Based Medical Systems (CBMS)*, 486–491 (IEEE, 2021).

[33] He, K., Zhang, X., Ren, S. & Sun, J. Delving deep into rectifiers: Surpassing human-level performance on imagenet classification. In *2015 IEEE International Conference on Computer Vision (ICCV)*, 1026–1034, 10.1109/ICCV.2015.123 (2015).

[34] Gao, S.-H. et al. Res2Net: A new multi-scale backbone architecture. *IEEE Trans. Pattern Anal. Mach. Intell.* **43**, 652–662. 10.1109/TPAMI.2019.2938758 (2021). 31484108 10.1109/TPAMI.2019.2938758

[35] Yu, F., Wang, D., Shelhamer, E. & Darrell, T. Deep Layer Aggregation. In *2018 IEEE/CVF Conference on Computer Vision and Pattern Recognition*, 2403–2412, 10.1109/CVPR.2018.00255 (IEEE, Salt Lake City, UT, 2018).

[36] Xie, S., Girshick, R., Dollár, P., Tu, Z. & He, K. Aggregated residual transformations for deep neural networks. In *2017 IEEE Conference on Computer Vision and Pattern Recognition (CVPR)*, 5987–5995, 10.1109/CVPR.2017.634 (2017).

[37] Dietterich, T. G. Ensemble methods in machine learning. In *International workshop on multiple classifier systems*, 1–15 (Springer, 2000).

[38] Yagis, E. et al. Effect of data leakage in brain MRI classification using 2d convolutional neural networks. *Sci. Rep.* **11**, 1–13 (2021). 34799630 10.1038/s41598-021-01681-w PMC8604922

[39] Consortium, T. M. *Project MONAI. Zenodo*10.5281/zenodo.4323059 (2020).

[40] Ginsburg, B. et al. Stochastic gradient methods with layer-wise adaptive moments for training of deep networks. *CoRR***abs/1905.11286** (2019). arXiv:1905.11286.

[41] Satia, I. et al. Assessing the accuracy and certainty in interpreting chest X-rays in the medical division. *Clin. Med.* **13**, 349–352. 10.7861/clinmedicine.13-4-349 (2013). 10.7861/clinmedicine.13-4-349 PMC4954299 23908502

[42] Ekpo, E., Egbe, N. & Akpan, B. Radiographers’ performance in chest X-ray interpretation: The Nigerian experience. *Br.J. Radiol.* **88**, 20150023 (2015). 25966290 10.1259/bjr.20150023 PMC4628526

[43] Waitt, C. J. et al. The effect of a tuberculosis chest X-ray image reference set on non-expert reader performance. *Eur. Radiol.* **23**, 2459–2468. 10.1007/s00330-013-2840-z (2013). 23652843 10.1007/s00330-013-2840-z PMC3738845

